# Homologous Recombination Deficiency in Skin Cancers: Prevalence and Clinical Implications of This Distinct Patient Cohort

**DOI:** 10.1200/PO-25-00923

**Published:** 2026-01-14

**Authors:** George Nassief, Tolulope Adeyelu, Andrew Elliott, Jordan Phillipps, Renee Morecroft, Alice Y. Zhou, Peter W. Szlosarek, Caroline Robert, Farah Abdulla, Ari Vanderwalde, Soo J. Park, David Chen, George Ansstas

**Affiliations:** ^1^ Division of Medical Oncology, Department of Medicine, Washington University in Saint Louis, Saint Louis, MO; ^2^ Caris Life Sciences, Phoenix, AZ; ^3^ Cancer Research UK Translational Oncology Laboratory, Barts and the London, London, United Kingdom; ^4^ Gustave Roussy Cancer Campus, Villejuif, France; ^5^ Division of Hematology/Oncology, Moores Cancer Center, University of California San Diego, La Jolla, CA; ^6^ Division of Dermatology, Department of Medicine, Washington University in Saint Louis, Saint Louis, MO

## Abstract

**PURPOSE:**

Homologous recombination deficiency (HRD) results in DNA instability in tumor cells and contributes to tumor pathogenesis. Although HRD-directed therapies are established in other cancers, their role in skin cancers remains unclear. Given the poor response to standard therapies in skin cancer subtypes like acral and mucosal melanoma, we aimed to characterize the prevalence of HRD across skin cancer subtypes, evaluate its correlation with immune checkpoint inhibitor (ICI) response biomarkers, and assess its prognostic relevance in patients treated with immunotherapy (IO).

**METHODS:**

A total of 2,508 patients with skin cancer underwent molecular profiling including whole-exome sequencing, whole-transcriptome sequencing, and immunohistochemistry. HRD status was defined by a high loss of heterozygosity (LOH-high) or mutations in homologous recombination repair (HRR) genes. Associations between HRD and established ICI biomarkers (tumor mutational burden, PD-L1, deficient mismatch repair/microsatellite instability-high, immune cell fractions, and transcriptomic signatures) were assessed. Survival outcomes on ICI therapy were assessed in cutaneous melanoma using insurance claims data.

**RESULTS:**

Overall, among the melanoma subtypes, mucosal and acral melanoma had a greater prevalence of LOH-high than cutaneous (30.9% *v* 13.1% *v* 8.3%, *P* < .001). Generally, LOH-high in skin cancer did not correlate with mutations in HRR genes. Additionally, there was no significant association in ICI response biomarkers and HRD among patients with skin cancers. Furthermore, HRD was not associated with a prognostic advantage following IO (hazard ratio, 0.981 [CI, 0.80 to 1.20]; *P* = .854).

**CONCLUSION:**

HRD defines a biologically distinct subset of skin cancers and is not predictive of ICI response or improved outcomes. The high prevalence of HRD in acral and mucosal melanoma highlights the need to investigate HRD-directed therapies strategies in this distinct cohort, such as poly (ADP-ribose) polymerase inhibitors or platinum-based therapies.

## INTRODUCTION

Several genomic biomarkers play a tumor-agnostic role in predicting response to immune checkpoint inhibitors (ICIs) and outcomes in melanoma.^1,2^ However, little is known about the role of homologous recombination deficiency (HRD) in melanoma pathogenesis and clinical outcomes. HRD is a state in which cells cannot repair DNA double-strand breaks using the homologous recombination repair (HRR) pathway.^3-5^

**CONTEXT**

**Key Objective**
To determine the prevalence of homologous recombination deficiency (HRD) across skin cancer subtypes, evaluate its relationship with immune checkpoint inhibitor (ICI) biomarkers, and assess its prognostic relevance in patients with skin cancer treated with immunotherapy (IO). This study provides the first large-scale genomic and transcriptomic assessment of HRD in melanoma and nonmelanoma skin cancers.
**Knowledge Generated**
Among 2,508 skin cancer samples, mucosal and acral melanomas demonstrated the highest prevalence of loss of heterozygosity–based HRD compared with cutaneous melanoma. HRD did not correlate with mutations in canonical homologous recombination repair genes or with established ICI biomarkers, including tumor mutational burden, PD-L1, and microsatellite instability-high/deficient mismatch repair status. HRD was not associated with improved survival following ICI therapy.
**Relevance**
These findings define HRD as a biologically distinct subset of skin cancers not predictive of additional IO benefit, supporting further evaluation of poly (ADP-ribose) polymerase inhibitors or platinum-based therapies in HRD-enriched acral and mucosal melanomas.

HRD status can be quantified by different scoring systems that assess genomic scarring rather than relying on alterations in HRR pathway genes, like *BRCA1/2*. One method generates a composite score derived from single or multiple genomic instability measures such as genome-wide loss of heterozygosity (LOH), telomeric allelic imbalance (TAI), and large-scale state transitions (LSTs).^6-9^

The main therapies used for HRD are poly (ADP-ribose) polymerase (PARP) inhibitors (PARPi) and platinum-based chemotherapies. PARPi exploit synthetic lethality in HRD tumors by blocking single-strand DNA repair, causing cell death in the context of defective homologous recombination. They are approved for HRD-associated ovarian, breast, prostate, and pancreatic cancers.^10-12^ However, their role in melanoma management remains unclear.

Early clinical data and case series have indicated HRD-based biomarkers, such as LOH and alterations in the HRR pathway, may help identify patients with melanoma who could benefit from PARPi, especially in those refractory to immunotherapy (IO).^6,13,14^ Thus, characterizing HRDness in melanoma and nonmelanoma skin cancers could offer insight as to how applicable PARPi could be used in this subset of patients.

In this report, we investigated the prevalence of LOH-based HRD across different skin cancer cohorts. HRD was defined based on the LOH status or presence of HRR gene mutation. Tumors with high LOH (>16%) or with HRR gene mutation were classified as HRD, whereas those with low LOH (<16%) or HRR gene wild-type status were classified as homologous recombination proficient (HRP). We further investigated the correlation between HRD and other established melanoma biomarkers to better understand mechanisms of tumor immunogenicity and immune evasion in this subcohort. In addition, we investigated correlations between the most common HRR pathway gene mutations and LOH-based HRD, aiming to evaluate the feasibility of using these alterations for establishing HRD status.

## METHODS

### Study Cohort

Our study examined male and female humans, and similar findings are reported for both sexes. Formalin-fixed paraffin-embedded (FFPE) samples from patients with skin cancer (n = 2,508) were submitted to a commercial Clinical Laboratory Improvement Amendments (CLIA)–certified laboratory for molecular profiling (Caris Life Sciences, Phoenix, AZ) and analyzed by whole-exome sequencing, whole-transcriptome sequencing (WTS), and immunohistochemistry (IHC). The study follows guidelines provided by the Declaration of Helsinki, Belmont Report, and US Common Rule. In accordance with compliance policy 45 code of federal regulations 46.101(b), this study was conducted using retrospective, deidentified clinical data, patient consent was not required, and the study was considered institutional review board exempt.

### DNA Next-Generation Sequencing (NGS)

Direct sequence analysis was conducted on genomic DNA isolated from micro-dissected FFPE samples using NextSeq or NovaSeq 6000 Platforms (Illumina, Inc, San Diego, CA). A custom SureSelect XT assay (Agilent Technologies, Santa Clara, CA) was used to enrich exonic regions 592 whole-gene targets. For tumor sample sequenced on NovaSeq 6000 platform, more than 700 clinically relevant genes were assessed. All variants were detected with >99% confidence on the basis of allele frequency and amplicon coverage, with an average sequencing depth of coverage of >500 and an analytic sensitivity threshold established of 5% for variant calling. TMB was measured by counting all nonsynonymous missense, nonsense, in-frame insertion/deletion, and frameshift mutations found per tumor that had not been previously described as germline alterations in dbSNP151, Genome Aggregation Database (gnomAD) databases, or benign variants identified by Caris's geneticists. High TMB was defined by a cutoff of ≥10 mutation/megabase (mut/MB) on the basis of the KEYNOTE-158 pembrolizumab trial, where it was shown that patients with ≥10 mut/MB had increased response rates compared with those with <10 mut/MB.^15^

Genomic LOH was calculated by analyzing approximately 250k single-nucleotide polymorphisms (SNPs) within segmented autosomal chromosomes. LOH-high/low was based on the percentage of all 552 segments with observed LOH (high, ≥16%; low <16%; if fewer than 3,000 SNPs were read, the test was reported as indeterminate). The Genomic Scar Score (GSS) incorporates both LOH and LST in an unweighted numerical sum. A positive GSS result means at least one of two possible criteria were met: *BRCA1/2* pathogenic or likely pathogenic mutations or a GSS ≥ 46. For cases with wild-type *BRCA1/2*, GSS results between 38 and 46 are called inconclusive, and scores below 38 are called negative.

### WTS

FFPE tissue sections mounted on glass slides underwent staining with nuclear fast red. Regions that contained a minimum of 10% tumor content were delineated for manual microdissection and subsequent mRNA extraction. WTS was executed using the Illumina NovaSeq platform (Illumina, Inc, San Diego, CA) along with the Agilent SureSelect Human All Exon V7 bait panel (Agilent Technologies, Santa Clara, CA), and the resulting data reported transcripts per million. Immune cell fraction was calculated using the quanTIseq pipeline, which employed deconvolution of bulk transcriptomic data.^16^ WTS data was also used to calculate interferon (IFN) gamma score as previously described.^17^

### IHC

IHC was conducted on complete sections of FFPE tissues mounted on glass slides. The slides underwent automated staining methods as directed by the manufacturer. These procedures were optimized and confirmed to meet the standards outlined by CLIA/certificate of accreditation and International Organization for Standarization. PD-L1 expression was determined using primary antibody SP142 (Spring Biosciences, Pleasanton, CA), with a positive threshold of ≥1+ stain intensity and ≥1% percentage of cells stained.

### Deficient Mismatch Repair/Microsatellite Instability-High (dMMR/MSI-H)

dMMR/MSI-H was determined by a combination of IHC using antibodies for MLH1 (M1 antibody), MSH2 (G2191129 antibody), MSH6 (44 antibody), and PMS2 (EPR3947 antibody) from Ventana Medical Systems (Tucson, AZ), and NGS. The outcomes from these platforms are mostly in agreement, as previously described.^18^ In instances where conflicting results emerged, the order of priority for determining the MSI/MMR status of the tumor was IHC followed by NGS.

### Outcome Data—CODEai

Real-world overall survival (rwOS) information was obtained from insurance claims data and calculated from time of treatment to last contact. Hazard ratio (HR) was calculated using the Cox proportional hazard models, and *P* values were calculated using the log-rank test with significance determined as *P* value of < .05.

### Statistics

Statistical analysis was performed using python packages—Pandas, NumPy, SciPy, or Graphpad prism (version 10.3.1). The comparison of continuous data was analyzed using the Mann-Whitney *U* test and categorical data was analyzed using the chi-square or Fisher exact test. Correction for multiple comparison was applied where appropriate using the Benjamini-Hochberg method to control for false discovery rate at a significance level of α = .05.

### Consent for Publication

All authors provided consent for the publication of this article.

## RESULTS

### Patient Characteristics

**FIG 1.**
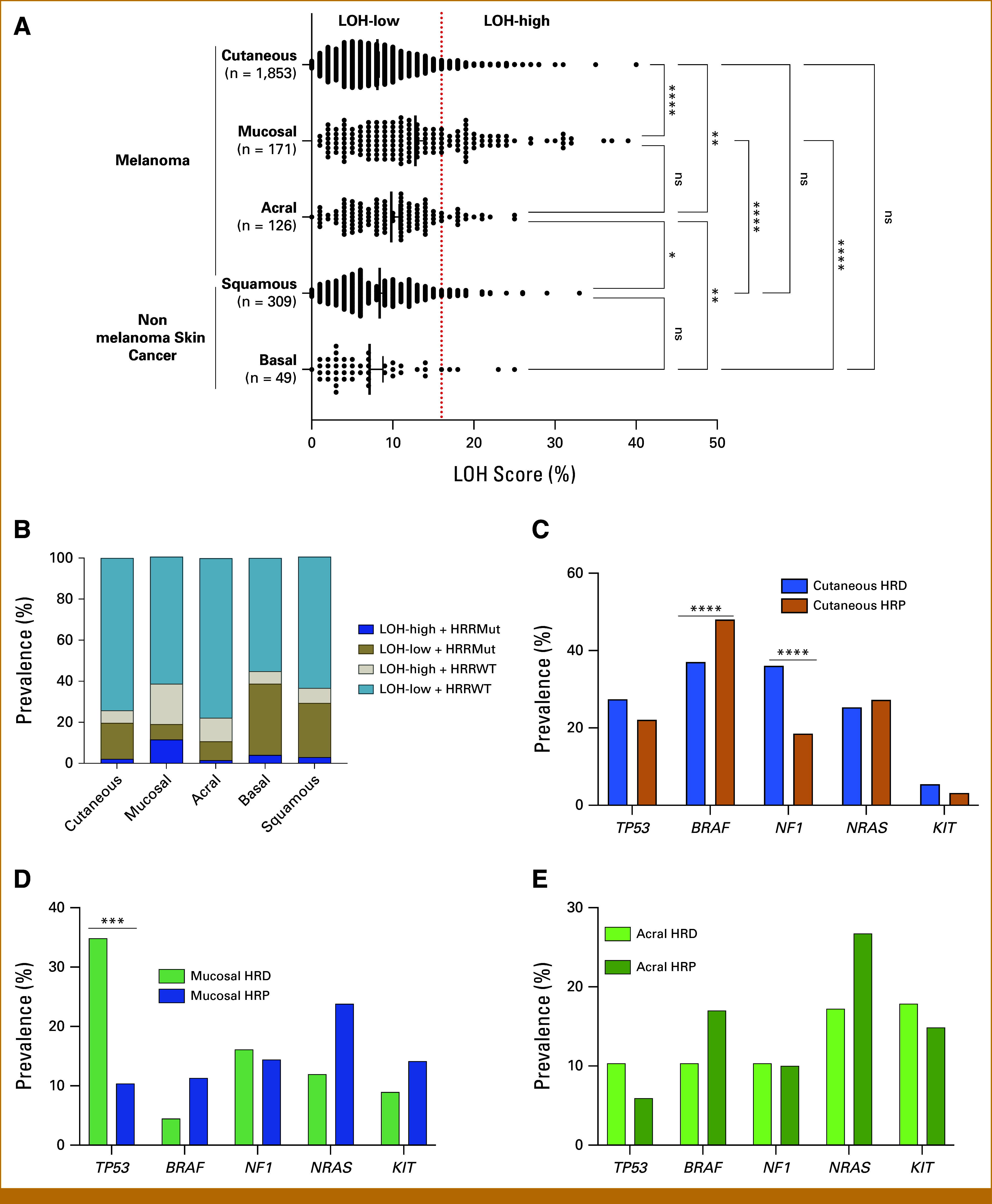
Distribution of LOH scores and prevalence of oncogene biomarkers across skin cancer samples. (A) Distribution of LOH scores across the skin cancer samples. Red line marks 16% threshold used to define LOH-high. (B) Comparison of the prevalence of 18 common HRR gene between LOH-high and LOH-low skin cancer cohorts. Comparison of the prevalence of common oncogene biomarkers between HRD and HRP (C) cutaneous melanoma, (D) mucosal melanoma, and (E) acral melanoma. *P < .05; **P < .01; P*** < .001; ****P < .0001. HRD, homologous recombination deficiency; HRP, homologous recombination proficient; HRR, homologous recombination repair; HRRMut, homologous recombination repair mutations; HRRWT, homologous recombination repair wildtype; LOH, loss of heterozygosity; ns, not significant.

**FIG A1.**
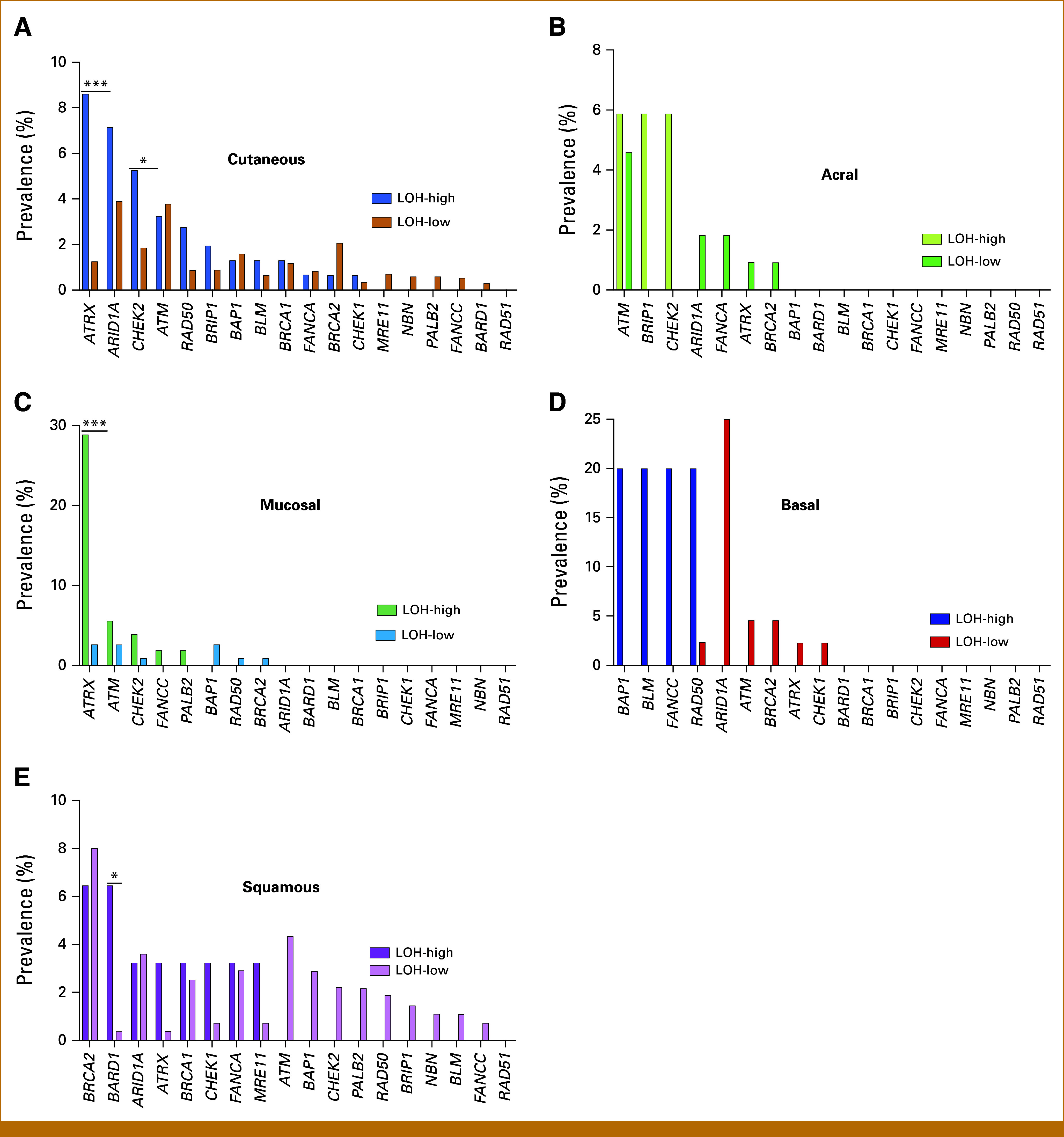
Negative correlation between HRR gene mutations and LOH-high score. HRR, homologous recombination repair; LOH, loss of heterozygosity.

A total of 2,508 patients (2,156 melanoma, 352 nonmelanoma) tested for LOH were examined (Tables 1 and 2). The cohort had a median biopsy age of 68 years (range, 5-89) and were 64.1% (n = 1,600) male. In melanoma cohorts, LOH-high was observed in 8.3% (n = 154) cutaneous samples, 13.1% (n = 17) acral, and 30.9% (n = 54) of mucosal samples. In nonmelanoma samples (n = 358), LOH-high was observed in 10% (n = 5) of basal cell carcinoma (BCC) and 10% (n = 31) of cutaneous squamous cell carcinoma (Fig 1A). A comparison of the distribution of LOH scores among the distinct melanoma and nonmelanoma samples demonstrated significantly higher LOH scores for both acral and mucosal melanoma compared with all other cohorts. The distribution of LOH scores was similar between acral and mucosal melanoma (Fig 1A). For samples with available GSS scores, we assessed the correlation between LOH score and GSS score. For all skin cancer cohorts, a strong positive correlation (R, 0.67-0.85, *P* < .01) existed between GSS and LOH (Appendix Fig A1).

**TABLE 1.** Clinicodemographic Breakdown for Melanoma HRD and HRP Patients (n = 2,158)

Characteristics of the Patients With Melanoma	Cutaneous HRD	Cutaneous HRP	Mucosal HRD	Mucosal HRP	Acral HRD	Acral HRP
Count, No.	478	1,375	67	106	29	101
Age, years, median (range), No.	70 (20-89)	67 (14-89)	70 (43-89)	69 (39-89)	63 (43-87)	69 (5-89)
Sex, No. (%)						
Male	311 (65.1)	905 (65.8)	14 (20.9)	32 (30.2)	17 (58.6)	55 (54.5)
Female	167 (34.9)	470 (34.2)	53 (79.1)	74 (69.8)	12 (41.4)	46 (45.5)
Race, No. (%)						
White	364 (76.2)	1,050 (76.4)	45 (67.2)	73 (76.8)	17 (58.6)	58 (57.4)
Black or African American	9 (1.9)	30 (2.2)	4 (6.0)	11 (11.6)	1 (3.4)	13 (12.9)
Asian or Pacific Islander	6 (1.3)	6 (0.4)	5 (7.5)	2 (2.1)	0 (0.0)	5 (5.0)
Other	18 (3.8)	69 (5.0)	3 (4.5)	1 (1.1)	2 (6.9)	4 (4.0)
Unknown	81 (16.9)	220 (16.0)	10 (14.9)	19 (17.9)	9 (31.0)	21 (20.8)
Ethnicity, No. (%)						
Not Hispanic or Latino	377 (78.9)	1,077 (78.3)	42 (62.7)	74 (77.9)	18 (62.1)	70 (69.3)
Hispanic or Latino	21 (4.4)	52 (3.8)	7 (10.4)	12 (12.6)	7 (24.1)	11 (10.9)
Unknown	80 (16.7)	246 (17.9)	18 (26.9)	20 (18.9)	4 (13.8)	20 (19.8)
Metastatic sites, No. (%)						
Primary	168 (35.1)	490 (35.6)	1 (1.5)	7 (6.6)	12 (41.4)	49 (48.5)
Brain mets	21 (4.4)	75 (5.5)	0 (0.0)	0 (0.0)	1 (3.4)	2 (2.0)
Lung mets	50 (10.5)	106 (7.7)	1 (1.5)	2 (1.9)	2 (6.9)	1 (1.0)
Lymph mets	78 (16.3)	269 (19.6)	7 (10.4)	5 (4.7)	6 (20.7)	21 (20.8)
Liver mets	25 (5.2)	43 (3.1)	3 (4.5)	3 (2.8)	0 (0.0)	2 (2.0)
Others	136 (28.5)	392 (28.5)	55 (82.1)	89 (84.0)	8 (27.6)	26 (25.7)
TMB						
Median TMB (range)	21 (0-417)	12 (0-209)	3 (1-49)	3 (1-35)	3 (1-9)	3 (0-22)

Abbreviations: HRD, homologous recombination deficiency; HRP, homologous recombination proficient; mets, metastasis; TMB, tumor mutational burden.

**TABLE 2.** Clinicodemographic Breakdown for Nonmelanoma HRD and HRP Patients (n = 352)

Characteristics of the Patients With Non-Melanoma Skin Cancers	Basal HRD	Basal HRP	Squamous HRD	Squamous HRP
Count, No.	22	27	111	192
Age, years, median (range), No.	62 (45-89)	61 (39-83)	72 (32-89)	74 (27-89)
Sex, No. (%)				
Male	15 (68.2)	19 (70.4)	85 (76.6)	147 (76.6)
Female	7 (31.8)	8 (29.6)	26 (23.4)	45 (23.4)
Race, No. (%)				
White	17 (77.3)	20 (74.1)	92 (82.8)	155 (80.7)
Black or African American	0 (0.0)	1 (3.7)	0 (0.0)	9 (4.7)
Asian or Pacific Islander	0 (0.0)	0 (0.0)	1 (0.9)	4 (2.1)
Other	3 (13.6)	1 (3.7)	4 (3.6)	4 (2.1)
Unknown	2 (9.1)	5 (18.5)	14 (12.6)	20 (10.4)
Ethnicity, No. (%)				
Not Hispanic or Latino	18 (81.8)	17 (62.9)	90 (81.1)	157 (81.8)
Hispanic or Latino	1 (4.5)	4 (14.8)	10 (9.0)	15 (7.8)
Unknown	3 (13.6)	6 (28.6)	11 (9.9)	20 (10.4)
Metastatic sites, No. (%)				
Primary	10 (45.5)	16 (59.3)	42 (37.8)	81 (42.2)
Brain mets	0 (0.0)	0 (0.0)	0 (0.0)	2 (1.0)
Lung mets	1 (4.5)	1 (3.7)	5 (4.5)	8 (4.2)
Lymph mets	1 (4.5)	0 (0.0)	20 (18.0)	28 (14.6)
Liver mets	0 (0.0)	0 (0.0)	2 (1.8)	3 (1.6)
Others	10 (45.5)	10 (37.0)	42 (37.8)	702 (36.5)
TMB				
Median TMB (range)	50.5 (12-185)	21 (4-75)	37.5 (3-268)	28.5 (0-227)

Abbreviations: HRD, homologous recombination deficiency; HRP, homologous recombination proficient; mets, metastasis; TMB, tumor mutational burden.

**FIG A2.**
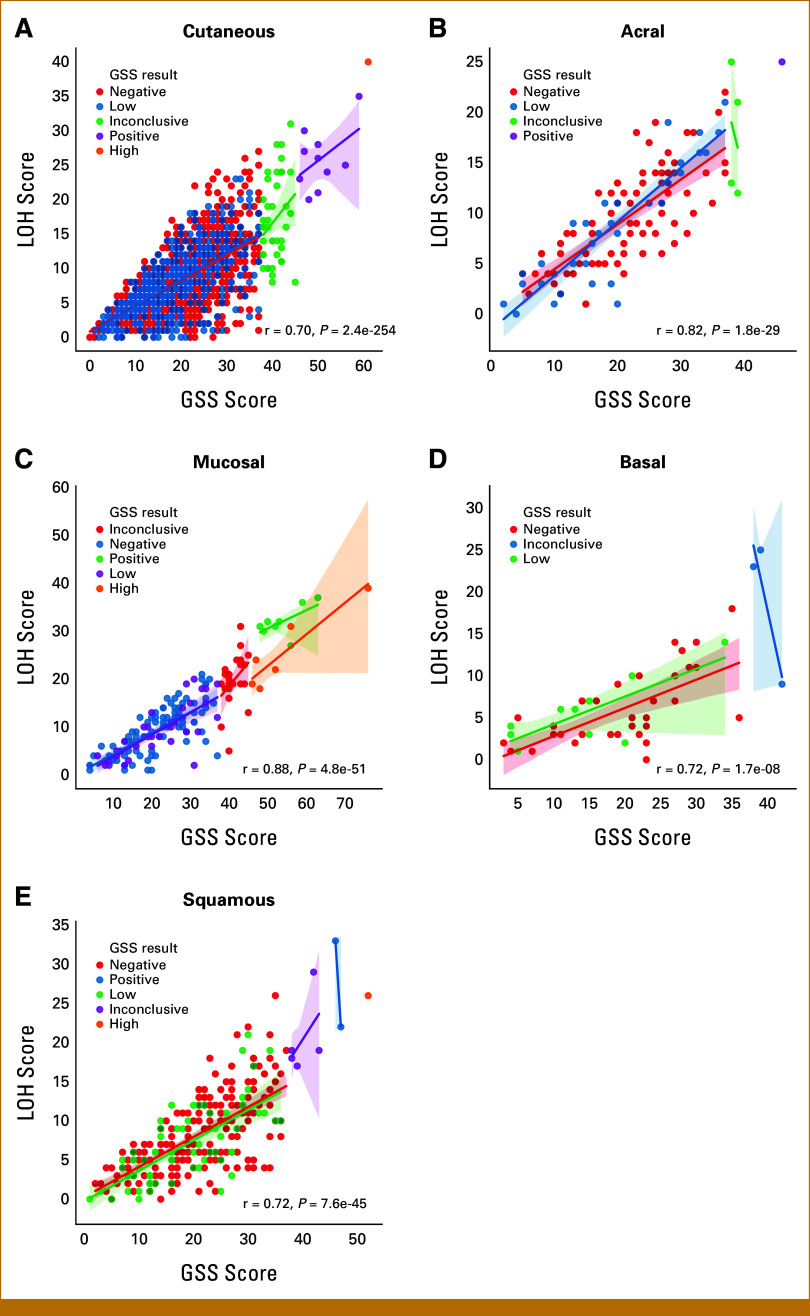
Positive correlation between GSS score and LOH score. GSS, Genomic Scar Score; LOH, loss of heterozygosity.

Additionally, the genomic LOH did not correlate with canonical HRR gene mutations, like *BRCA1/2*. In cutaneous melanoma, mutations in *BRCA1/2* were not statistically different between LOH-high and LOH-low (*BRCA1*: 1.30% *v* 1.12%, *P* = .705; *BRCA2*: 0.65% *v* 2.07%, *P* = .358). However, genes like *ATRX* (8.61% *v* 1.252, *P* < .01) and *CHEK2* (5.26% *v* 1.86%, *P* = .013) were more frequently mutated in LOH-high samples compared with LOH-low (Appendix Figs A2A-A2E).

To, therefore, comprehensively capture the HRD in this cohort, we included patients with both HRR gene mutations and LOH-high status. Overall, in the melanoma cohorts, HRD was observed in 25.9% cutaneous (n = 478), 38.3% mucosal (n = 67), and 22.3% acral (n = 29) melanoma. While in the nonmelanoma cohorts, HRD was observed in 44.89% basal (n = 22) and 36.6% squamous cell carcinoma (n = 111; Tables 1 and 2; Fig 1B).

### Correlation Between Melanoma Genetic Biomarkers and HRD Status

For the melanoma cohorts, we evaluated differences in mutation of known biomarkers based on their HRD status. Cutaneous melanoma samples with HRD had significantly higher prevalence of *NF1* mutation (36.06% *v* 18.5%, *P* < .001) and significantly lower prevalence of V-RAF murine sarcoma viral oncogene homolog B (*BRAF*) mutation (48.0% *v* 37.03%, *P* < .01; Fig 1C). In the mucosal melanoma cohort, HRD was associated with significant higher *TP53* mutation (34.85% *v* 10.38%, *P* < .01) with no association with *BRAF* (4.48% *v* 11.32%, *P* = .752) and *NRAS* (11.94% *v* 23.81%, *P* = .475) mutations (Fig 1D). In acral melanoma, there was no difference in the *TP53*, *BRAF*, *NF1*, *NRAS*, and *KIT* mutation rates based on the HRD status (Fig 1E).

### Correlation Between Common Immunogenicity Biomarkers and HRD Status

**FIG 2.**
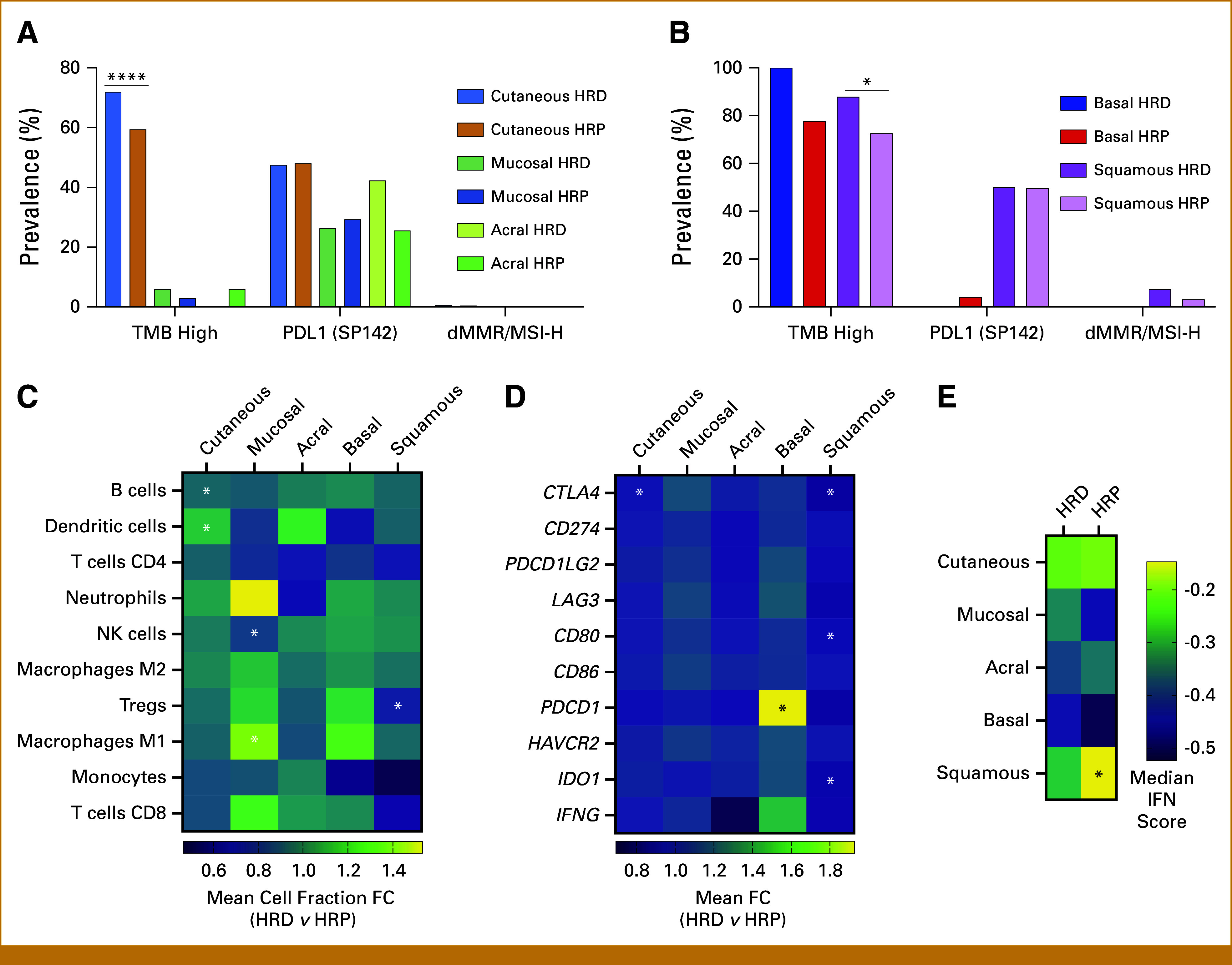
Potential ICI response markers in skin cancer samples. (A) Prevalence of TMB-high, dMMR/MSI-H, and PD-L1 in HRD and HRP melanoma skin cancer samples. (B) Prevalence of TMB-high, dMMR/MSI-H, and PD-L1 in HRD and HRP nonmelanoma skin cancer samples. (C) Comparison of the FC in TME cell composition changes between HRD and HRP skin cancer cohorts. (D) Comparison of the FC in gene mutations involved in ICI therapy response between HRD and HRP skin cancer cohorts. (E) Comparison of the FC in IFN gamma levels between HRD and HRP skin cancer cohorts. *P < .05; ****P < .0001. dMMR, deficient mismatch repair; FC, fold change; HRD, homologous recombination deficiency; HRP, homologous recombination proficient; ICI, immune checkpoint inhibitor; IFN, interferon; MSI-H, microsatellite instability-high; NK, natural killer; TMB, tumor mutational burden; TME, tumor microenvironment.

The prevalence of IO response biomarkers was compared between HRD and HRP skin cancer cohorts. TMB-high prevalence was higher in HRD cutaneous melanoma (71.9% *v* 59.4%, *P* < .001) and HRD squamous cell carcinoma (88.0% *v* 72.6%, *P* = .04). The prevalence of TMB-high was not statistically different between HRD and HRP in mucosal (5.97% *v* 2.88%, *P* = .998), acral (0.00% *v* 5.94%, *P* = .998), or BCC (100.00% *v* 77.78%, *P* = .334). However, there was no difference in the prevalence rates of other biomarkers such as PD-L1 and dMMR/MSI-H, based on the HRD status in both melanoma and nonmelanoma cohorts (Figs 2A and 2B).

We further evaluated the correlation between the HRDness and tumor microenvironment (TME) using quanTIseq, an immune deconvolution approach that estimates immune cell fractions using RNA-seq data. For cutaneous melanoma cohorts, samples with HRD showed significant elevation of dendritic cells (mean fold change [FC] = 1.11, *P* = .041) with significantly less enrichment in B cells (FC = 0.91, *P* = .038). We also observed a trend of lower monocytes (FC = 0.84, *P* = .077) and Tregs (FC = 0.93, *P* = .078) in the HRD cutaneous melanoma samples. For mucosal melanoma, HRD was associated with lower natural killer cell (FC = 0.81, *P* < .001) and higher M1-polarized macrophages cell fractions (FC = 1.38, *P* < .001). In acral melanoma cohorts, the immune fraction within the TME was similar between HRD and HRP samples (Fig 2C).

In nonmelanoma cohorts, squamous cell carcinoma with HRD had significantly lower Tregs (FC = 0.61, *P* < .001) compared with those with HRP (Fig 2C). Additionally, no significant difference in the immune fraction within the basal cell skin cancer TME was observed.

Furthermore, we evaluated the expression levels of IO target genes across melanoma and nonmelanoma skin cancer with HRD. HRD in nonmelanoma skin cancers was associated with lower expression of IO target genes. In squamous cell carcinoma, CD80 (FC = 0.88, *P* = .014), CTLA4 (FC = 0.80, *P* < .01), and IDO1 (FC = 0.84, *P* = .020) expression was lower in HRD cohorts compared with HRP. In addition, in BCC, the level of PD1 gene (FC = 1.92, *P* < .01) was significantly higher in HRD samples compared with those with HRP. However, in melanoma subtypes, HRP and HRD did not associate with IO targets (Fig 2D). Meanwhile, the IO-related transcriptomic biomarker IFN gamma score was not statistically different between HRD samples and HRP samples across all skin cancer subtypes except in squamous cell carcinoma where HRD shows a significant decrease in IFN gamma scores (−0.29 *v* −0.15, *P* = .0015; Fig 2E).

### Survival Outcomes Appear to Be Comparable Between LOH-High and LOH-Low Patients

**FIG 3.**
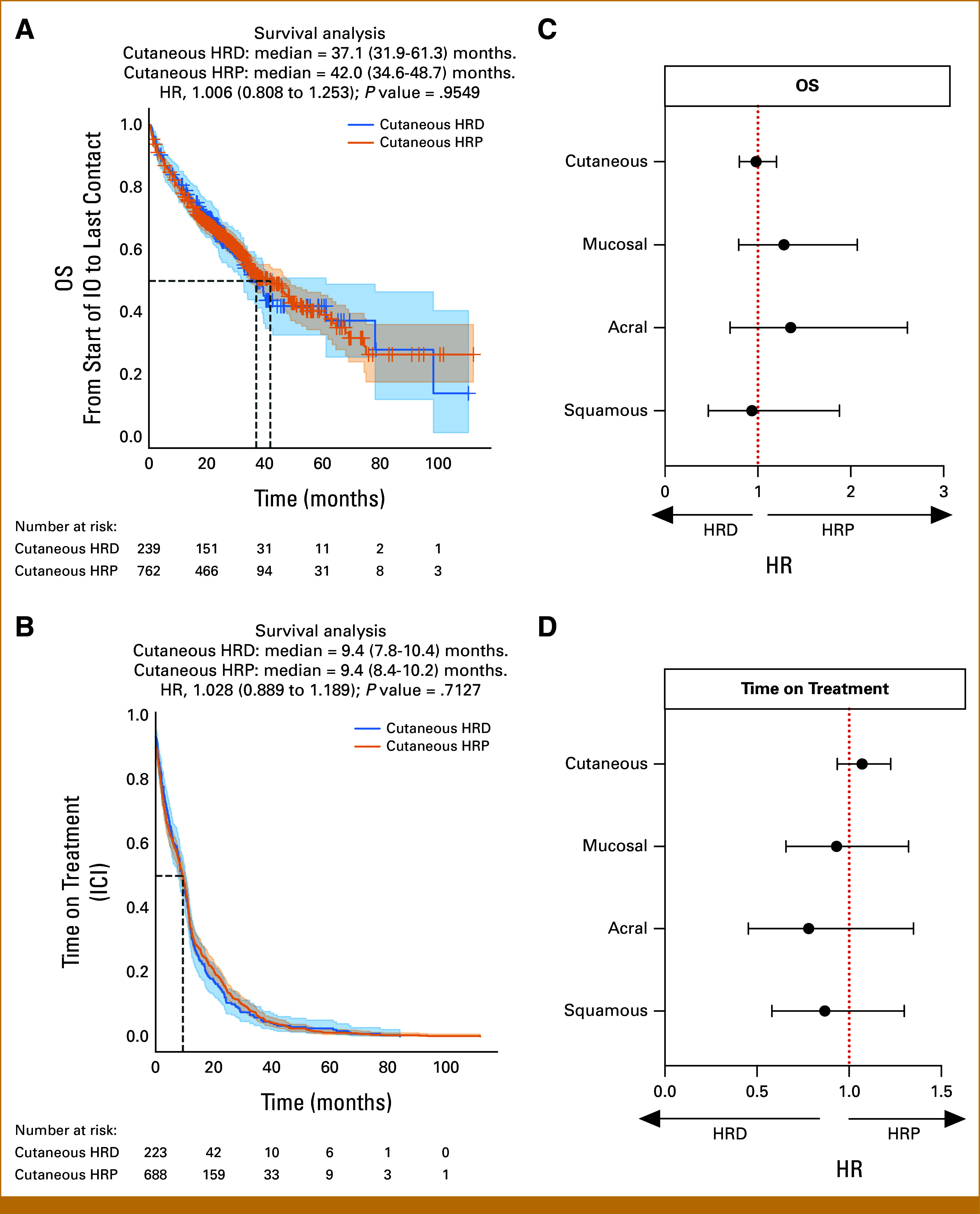
Real-world survival analysis on cutaneous melanoma cohorts. (A) Kaplan-Meier curve analysis of the OS in cutaneous melanoma in HRP and HRD patients from time of start of IO to last contact. (C) Kaplan-Meier curve analysis of the time on treatment in cutaneous melanoma in HRP and HRD patients. (B) HR of OS comparing HRP and HRD skin cancer cohorts. (D) HR of time on treatment comparing HRP and HRD skin cancer cohorts. HR, hazard ratio; HRD, homologous recombination deficiency; HRP, homologous recombination proficient; ICI, immune checkpoint inhibitor; IO, immunotherapy; OS, overall survival.

Analysis of rwOS demonstrates no significant difference in outcomes in cutaneous melanoma between HRD and HRP patients from the time of start of IO therapy to last contact (HR, 0.98 [CI, 0.80 to 1.20]; *P* = .853; Fig 3A) and time on treatment with IO therapy (HR, 1.07 [CI, 0.94 to 1.23]; *P* = .326; Fig 3B). In addition, our analysis demonstrates no significant difference in outcomes in nonmelanoma skin cancer considering either rwOS from start of IO to last contact (Fig 3C) or time on IO (Fig 3D).

## DISCUSSION

To our knowledge, this study represents the first comprehensive investigation to evaluate the prevalence of HRDness in melanoma and nonmelanoma skin cancer. We observed a significantly higher number of patients with LOH-based HRD in acral and mucosal melanoma compared with other melanoma subtypes. Our findings indicate that there is no significant correlation between predictive biomarkers for ICI response (PD-L1, TMB high, and T-cell inflamed^19-22^) and HRD compared with HRP in skin cancer. Thus, our results suggest that HRD in skin cancer defines a biologically distinct subgroup, which may benefit from other treatment modalities such as PARPi and platinum-based therapies.

Importantly, there was no difference in clinical outcomes following IO in patients with HRD compared with HRP in cutaneous melanoma, indicating that HRD patients do not exhibit more benefit from ICI therapy compared with HRP patients. Similarly, another study found no correlation between DNA-based HRD and IO response quantified through progression-free survival (PFS) and OS in metastatic clear cell renal cell carcinoma.^23^ This corresponds well with our findings and highlights how HRD patients with skin cancer may lack the IO response advantage compared with HRP found in other solid tumors, limiting therapeutic options for these patients.

Importantly, no correlation between canonical HRR mutations and HRD in patients with skin cancers was observed. A pan-cancer analysis done on 33 cancers including cutaneous melanoma and uveal melanoma observed significantly higher HRDsum scores calculated based on LOH, LST, and TAI in patients with 22 different HRR mutations including *BRCA1* and *BRCA2*.^24^ However, when broken down by cancer type, there was no association between HRDsum scores and a wider array of 142 HRR gene mutations in cutaneous melanoma and uveal melanoma.^24^ These results are significant to the nature of our work and confirm the unique profile patients with skin cancers have in terms of HRR mutations not being well predictive of HRD status.

In this context, identifying alternative molecular markers such as HRD in acral or mucosal melanoma is critical, given how these tumors have low TMB and BRAF V600 alterations, and, therefore, these tumors have not benefited substantially from the recent advances in ICIs and BRAF/mitogen-activated protein kinase (MEK) inhibitors. Our study showed a high prevalence of LOH in mucosal and acral melanoma compared with cutaneous melanoma. Indeed, acral and mucosal melanomas constitute a high medical need, and our results strongly suggest that these cancers should be more extensively screened for HRD, given the potential for response to PARPi.

We previously reported an advanced acral melanoma case with an *EMSY* gene amplification, which is not part of the traditional HRR gene mutations but is known to suppress HRR, to demonstrate a complete response to PARPi.^25^ Similarly, reports of increased platinum sensitivity, a well-known characteristic of HRD-deficient tumors, particularly in mucosal and acral melanoma with improved survival outcomes deserve further scrutiny in the context of our present study.^26-28^ Thus, the results from our study are critical in potentially guiding the development of HRD-directed therapeutic strategies, including PARPi and platinum-based regimens, and in establishing HRD as a biomarker to refine patient selection and expand treatment options for acral and mucosal melanoma.

In a recent phase II study in patients with advanced melanoma who harbored an HR mutation, niraparib, a PARPi, demonstrated a disease control rate of 64% and a median PFS of 16 weeks in patients who progressed on PD-1 blockade or BRAF/MEK inhibition. More than 70% of the patients recruited were of melanoma subtypes less likely to respond to ICI, such as acral, mucosal, and uveal primary melanoma.^29^ Although our study emphasizes that HRR gene mutations may not be fully representative of HRD status, the results of this study are still promising in the context of our work in highlighting how patients with advanced skin cancers may not fully benefit from ICI therapy alone. More studies are therefore needed to focus on ICI-nonresponsive, HRD-enriched subtypes like acral and mucosal melanoma and prospectively test whether HRDness stratifies benefit from alternative regimens like PARPi in a biomarker-driven design.

Our study comes with several limitations. We provided a surrogate for HRD using an LOH-based with a HRR mutation approach. However, a GSS, although correlated with LOH, may be a more comprehensive way to quantify HRD, but this was not available for our study. Additionally, our clinical response data were obtained from insurance claims data which has its own limitations. For instance, we reported surrogate for OS as time from start of IO therapy (ipilumumab, nivolumab, or pembrolizumab) to last contact. This analysis lacks granular data regarding radiographic response to treatment or reasons why treatment was discontinued. Additionally, owing to the retrospective nature of our study, we were unable to control for potential confounding clinical variables in our survival analysis. Nonetheless, future studies are needed to address the inherent limitations presented in our analysis.

In conclusion, to our knowledge, this study presents the largest and most comprehensive evaluation of HRD in skin cancers to date. Our results demonstrate that HRD in skin cancers defines a biologically distinct subgroup that lacks established biomarkers of immune privilege and shows no survival benefit from ICI. Our findings of a high prevalence of LOH in acral and mucosal melanoma subtypes that are known to demonstrate poor ICI response, highlights HRD as a promising biomarker with other potential therapeutic relevance. Future prospective studies incorporating GSSs and integrating detailed clinical response data are essential to validate HRD as a predictive biomarker and to accelerate the development of HRD-directed therapeutic strategies that could expand treatment options for this high-need patient population.
